# Course induced dexterity development and cerebellar grey matter growth of dentistry students: a randomised trial

**DOI:** 10.1038/s41598-021-84549-3

**Published:** 2021-03-17

**Authors:** Benedek Siman, Jozsef Janszky, Gabor Perlaki, Adrien Fazekas, Balazs Sandor, Krisztian Katona, Gyula Marada, Ildiko Szanto

**Affiliations:** 1 Department of Dentistry, Medical School, University of Pecs, Dischka Gyozo street 5, Pecs, 7621 Hungary; 2 Department of Neurology, Medical School, University of Pecs, Pecs, 7623 Hungary; 3 MTA-PTE Clinical Neuroscience MR Research Group, Pecs, 7623 Hungary

**Keywords:** Health care, Dentistry, Dental education, Extended skills training in dentistry

## Abstract

This study primarily focuses on the assessment of dentistry students’ improvement of manual skills resulting from their participation in courses. We aimed to prove that systematic manual skills development significantly improves dexterity. We hypothesized that the dexterity training regimen improves manual dexterity demonstrated by the HAM-Man (Hamburg Assessment Test for Medicine-Manual Dexterity) test scores and CGM (cerebellar grey matter) growth. Thirty volunteers were randomly divided into two equal groups (study and control). Firstly, volunteers were examined by the HAM-Man test and baseline MRI scans. Afterwards, a manual skills development course was launched for the “study group”. Secondly, all the manual skills of the students were evaluated longitudinally, by the HAM-Man test. Simultaneously, the follow-up MRI scans were taken to observe morphologic changes in the cerebellum. The Wilcoxon signed-rank test and Student Paired *t*-test were used for statistical analyses. Value p < 0.05 was considered significant. After the training, significant growth of CGM as well as improvement on manual skill assessment tests, were found in the study group. Training courses are suitable for preparing students with low levels of dexterity for performing demanding tasks. The improvement is demonstrable by a wire bending test and by bilateral CGM enlargement as well.

## Introduction

Manual dexterity is defined by the American Dental Association, as “the ability to use your hands in a skillful, coordinated way, to grasp and manipulate objects and demonstrate small, precise movements”^1^. In order to perform dental procedures, a dentist must be able to work with precision in an extremely small space. Manual dexterity necessary for the dental profession has not been defined precisely up till now. Studies explore the importance of manual, spatial, stereopsis, hand–eye coordination, cognitive, perception, handwriting, and drawing skills in the field of dentistry^1–8^.

While some university admission tests include the evaluation of spatial and manual abilities, others do not attach too much importance to them^9^. In an extreme case, the aim of such tests were identified as spotting unsuitable students (1 or 2 per year in classes of 45 undergraduates)^10^. The literature on the evaluation of these ability aptitudes confirms that no widely accepted opinion exists^2^. The lack of consensus may be explained by varied training systems and different university curricula.

Based on the conclusions of a comprehensive study^11^, we assume that manual aptitude tests should be used mainly for assessing first-year students’ skills. They need more careful, attentive, and monitored training, rather than being neglected right from the beginning of the entrance examination^11^.

In Hungary, there is no specified admission system for dental study courses. The selection is carried out primarily by Grade Point Averages (GPA). Therefore, instructors of dental practices at our university recognise or identify the students’ abilities only during preclinical practices in the fifth semester. Our vision, is that it is necessary to develop manual skills during the basic modules, prior before preclinical dental studies. Giuliani et al. proved that dental students are able to acquire the necessary dental skills after adequate practical skills development^11^.

As the human brain is able to adapt to different environmental stimuli and acquire new skills, cerebellar neuroplasticity can facilitate several skills learning^12^. This has already been described in a neuro-imaging study where the head chef's cerebellar volume was compared to that of the non-experts^12^.

The role of the cerebellum was highlighted in hand–eye coordination^13,14^, handwriting^15^, drawing^16^, stereopsis^17^, mental rotation^18^, spatial and cognitive tasks^19^, intentional tremor^20^, fine movement and coordination control^21^, motor learning^22^, perceptual processes^23^ and learning fine dexterous skills such as playing the keyboard^24^. Plasticity of the human brain in response to learning and experience has been reported by several earlier studies^25^. Cerebellar functions are well researched in their roles of balance, speech, eye and limb movements. These are often associated with patients with cerebellar disorders^26^. Functional neuroimaging studies (MRI and PET- Positron Emission Tomography) have examined the function of the cerebellum in motor control, automation, and motor skills acquisition^27^. In relation to reach-to-grasp movement evaluated by MRI and PET studies, it was found that the cerebellum has an important role in the adaptive modification of motor output and sensor-motor coordination^28–31^. The cerebellum is also essential for processing visual feedback during visually controlled movements and predicting the sensory interpretation of motoric events^30^. Recent transcranial direct current stimulation (tDCS) studies suggest that the cerebellum has a critical role in both the acquisition and retention of motor skills^32–34^. Moreover, the cerebellum is a highly plastic brain region^35,36^, which is related to the majority of skills shown to be important in the field of dentistry. These findings allow us to conclude that strengthening dental skills by a dexterity training course, may induce changes in the structure of the cerebellum.

Doyon and Ungerleider’s model of brain plasticity changes, describes an important role of the cerebellum during new motor skills learning^36^. Their theory evolved along two distinct pathways: the corticostriatal and the corticocerebellar systems. During motor learning, this model describes both behaviours (motor sequence learning and motor adaptation) and the phases of the motor learning processes (like fast learning, slow learning, consolidation, automatization and finally retention)^37,38^. In the case we present here, this motor learning process itself emphasises the improvement of manual dexterity.

Based on Doyon and Ungerleider’s model^36^, we hypothesise that training course exercises enhance motor adaptation. In the model design, the cerebellum (corticocerebellar system) play a decisive role in slow learning and in the consolidation phase of motor adaptation^39^. The reach of these phases is detectable by CGM growth and HAM-MAN test scores improvement.

Students’ training can be categorized as slow learning, which means that students could reach the final phases, such as consolidation, or automatization. By the end of the course, students reach the consolidation phase of learning, the stage where the cerebellum plays a significant role.

### The aim of the study

This study aims to demonstrate how our specially developed training course improves the manual dexterity of students trained.

We hypothesised that after developing the training system, we can observe and describe the improvement of manual dexterity, which is controlled and evidenced by the HAM-Man dexterity test and the CGM enlargement.

## Materials and methods

### Study design

The study was approved by the local ethical committee of the University of Pécs (No. 5495). All procedures were performed in accordance with the relevant guidelines and regulations, and consent was obtained from all participants. Our trial was parallel, and the allocation ratio was 50–50%.

At the beginning of the fall semester, the first HAM-Man tests and cerebellar MRI examination were carried out in both groups (first-time point: pre-training stage).

Starting on the second week of the semester, a targeted training course was weekly organized for the study group over a period of 10 weeks. The control group did not participate in any official training courses.

**Figure 1:**
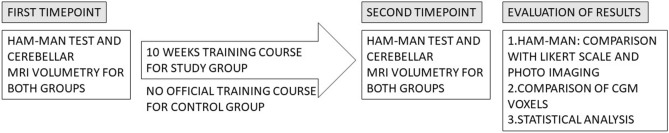
The study design.

After the training period, the HAM-Man test and the MRI imaging were repeatedly carried out on both groups (second-time point: post-training stage). Following the second examination, all evaluations between both time points were compared and analyzed statistically. For details of the study design see Fig. 1.

### Participants

All freshmen dental students were offered to participate in the study. A total of 55 students of the University Medical School of Pécs registered as volunteers of the study. We excluded left-handed students and those playing musical instruments, as well as senior medical students. Finally 30 (right-handed) freshman dentistry students were selected as participants in our study. We divided the participants into two groups by block randomisation. Concealment of allocation was implemented by SNOSE technique^40,41^. Fifteen students were involved in both groups. The first group, called “the study group” who were selected to train in our course, (average age ± standard deviation: 20.80 ± 2.06 years, male/female ratio: 5/10). The second group, called “the control group” also included 15 students as control persons, who did not receive any special training, (average age ± standard deviation: 20.06 ± 0.69 years, male/female ratio: 4/11). The students were involved voluntarily and gave their written consent to participate. During the whole study, the same volunteers participated, there were no exclusion procedure following the start.

### The details of the training course

Over the 10-week training period, a 60-min practice per week was held for the study group. The topic areas that the course included were as follows: freehand drawing, instructed drawing, picture magnification, drawing of selected picture particles, wax carving of geometric shapes and tooth shapes, soft wax modelling, wire bending after visual 2D instruction, and finally insertion of spikes within a limited time. The common course aimed to develop students’ eye-sketching, depth perception, hand–eye coordination, fine motor and drawing skills as well as the demand for self-improvement. The syllabus was developed by a ceramic artist and a dental technician, who conducted the practices together.

### The details of HAM-Man test

The HAM-Man test is meant to measure the manual skills of dentistry students^42,43^. The test was conducted to determine the manual dexterity of students, measured at the beginning and at the end of the training course. Manual skills were examined with a wire-bending test according to the original HAM-Man test^42^. The method required bending three shapes with a pair of pliers within a limited period of time (45 min).

**Figure 2:**
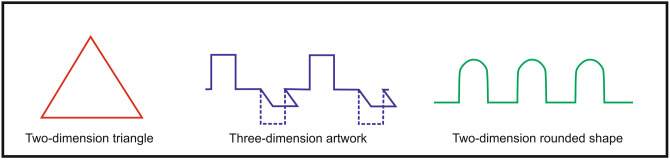
The HAM-Man wire bending shapes (made by I. Szanto).

Each student was instructed to bend three wire shapes in a laboratory environment. Each student received their own numbered envelope that contained a pair of crampon pliers, three pieces of chromium wires (150 mm length and 0.8 mm diameter), and the instructive drawings of three wire shapes to be bent. The first shape was an equilateral triangle, the second one was a three-dimensional bent shape and the third one was a two-dimensional shape that contained three waveforms (Fig. 2).

#### The assessment of bent wires

In addition to the original evaluation (Likert scale) we supplemented the assessment of the bent wires to support objectivity. The accuracy of the fitting was measured in two ways: in a semi-subjective and an objective manner.

#### The semi-subjective assessment method

The wire bending HAM-Man test was first assessed with the Likert scale. Two independent raters assessed all of the three bent wires on a Likert scale from 0 (least similar shape) to 6 (almost identical shape). Judges were selected from our Clinic were provided with training instructions. They evaluated blindly each wire, then they discussed their judgement, and gave an average grade of their consequential results. This evaluation is a “subjective” method, relying on the raters’ individual opinions.

#### The objective assessment method

**Figure 3:**
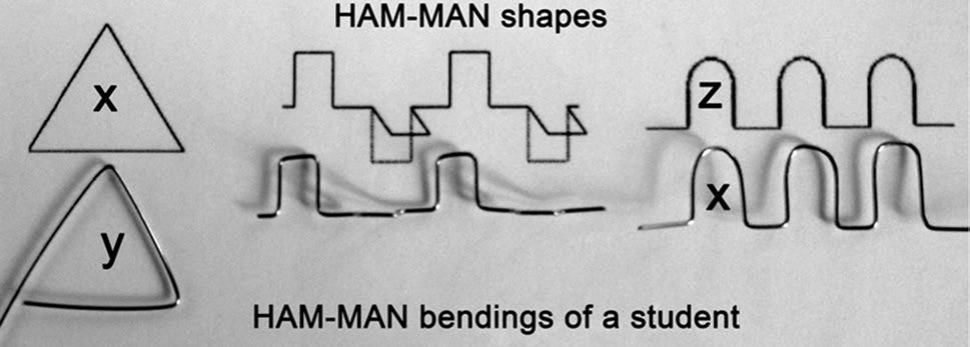
The photo used for pixel calculation (made by B. Siman).

The objective evaluation included the comparison of pixel numbers. We took a photo of each instructive drawings together with the bent wires. The photos were taken at the same time, in the same settings, using a tripod. We focused on the two-dimensional products, because the surface measurement in that cases were simplier The two-dimensional measurable areas in the photos are below the first wave, and inside the total triangle, which are signed with letters: x, y, z (Fig. 3). The areas in the photos were analysed by the software both on the instruction picture and also on the wire picture. The pixels of that areas were counted using a photo editing software (Adobe Photoshop CS6 Extended, Adobe Inc. San José, California). We performed a computer assisted evaluation of all photos, and compared the original drawings, and the wire shapes of triangles and waves regarding the pixel differences: the positive or negative distance between the instructive shapes and the two-dimensional bent wires were detected. The mean deviations were calculated mathematically.

### The details of MRI acquisition

All of the baseline and follow-up images were acquired using the same 3T MRI scanner (MAGNETOM Trio, Siemens AG, Erlangen, Germany) with a 12-channel head coil. T1-weighted three-dimensional MPRAGE scans with the following parameters were acquired: TR/TI/TE = 2530/1100/3.37 ms; flip angle = 7°; 176 sagittal slices; slice thickness = 1 mm; FOV = 256 mm × 256 mm; matrix size = 256 × 256; receiver bandwidth = 200 Hz/pixel. Visual analysis of the images identified no brain abnormalities.

The CGM volumes (measured in mm^3^) of the left and right cerebellum were estimated in both groups before and after the training course. Paired t-test was applied in both groups.

The CGM volumes were obtained from the T1-weighted MPRAGE images using the longitudinal stream of FreeSurfer v5.3.0 image analysis suite (https://surfer.nmr.mgh.harvard.edu/). All of the images were first evaluated using the standard cross-sectional processing stream of the software. The technical details are described in previous methodological publications^44,45^ and on the FreeSurferWiki page (https://surfer.nmr.mgh.harvard.edu/fswiki, https://surfer.nmr.mgh.harvard.edu/fswiki/LongitudinalEdits).

Briefly, the processing includes intensity normalization^46^, affine registration to the MNI305 template^47^, a further intensity normalization and the removal of non-brain tissue using a hybrid watershed/surface deformation procedure^48^, linear and non-linear registrations to FreeSurfer’s default Gaussian classifier array (GCA) atlas, and finally, automated volumetric labeling^44,48^. This labeling is based on both subject-specific measured values and a subject-independent probabilistic atlas (i.e. GCA atlas).

Next, the baseline and follow-up images of each subject were processed using the longitudinal stream, which increases the reliability by creating a robust within-subject template to initialize both time points^49,50^. The estimates of CGM volumes were derived from the longitudinal pipeline.

Quality control was performed throughout the automatic processing stream. Error correction was performed when necessary, and is based on the recommended workflow. All scans were evaluated using the same operating system, employing the same software version to avoid potential mistakes^51^.

### Statistical analyses

All data were collected and administered by the main author The Wilcoxon signed-rank test was used to measure the differences at wire bent tests (IBM SPSS 24.0 software) between the two time points. After normality testing, CGM data were compared between the two time points via Student Paired *t*-test (IBM SPSS 24.0) for both groups by evaluating the MRI results. A probability value p < 0.05 was considered significant.

## Results

### Manual dexterity results

The results of manual dexterity tests of the trained (study) group showed significant elevation, while there was no notable increase in the control group (Table 1a,b).

**Table 1:** The results of the development of manual ability, study group (a) n = 15, control group (b) n = 15.

Manual ability variable changes (between time point 2 and 1)	Median range	SD	25% percentile	75% percentile	p^a^
**(a) Study group (n = 15)**					
Likert evaluation	1 (− 1 to 3)	1.361	0	2.5	0.0148
Pixel difference calculation of wave bending	− 180 (− 1219 to 442)	368.1	− 376	− 79	0.0143
Pixel difference calculation of triangle bending	− 158 (− 6131 to 162)	1830	− 847	− 43	0.0118
**(b) Control group (n = 15)**					
Likert evaluation	0 (− 1.5 to 1)	0.9297	− 1	1	0.6138
Pixel difference calculation of wave bending	115 − 279 to 1045)	340.1	− 56	440	0.073
Pixel difference calculation of triangle bending	214 (− 984 to 1214)	535.5	32	495	0.1205

The results of the development of manual dexterity variable changes between time points 2 and 1 (after the training course and before it). Median, range, standard deviation (SD), interquartile range (25 and 75%) and probability (p) values are given for Likert evaluation and for the pixel difference of the two bending types (wave and triangle).

^a^Wilcoxon signed-rank test.

Tables 1a,b show the manual dexterity changes between the baseline and follow-up measurements of the two groups evaluated with subjective (Likert) and objective (pixel differences) methods. The words “wave” and “triangle” refer to the 2-dimensional bent shapes (Fig. 2). Negative change of median range of pixel differences indicates smaller pixel difference between the instructive sample shapes and bent wires at the second time point compared to the first time point, which counts as a rising tendency of dexterity.

**Figure 4:**
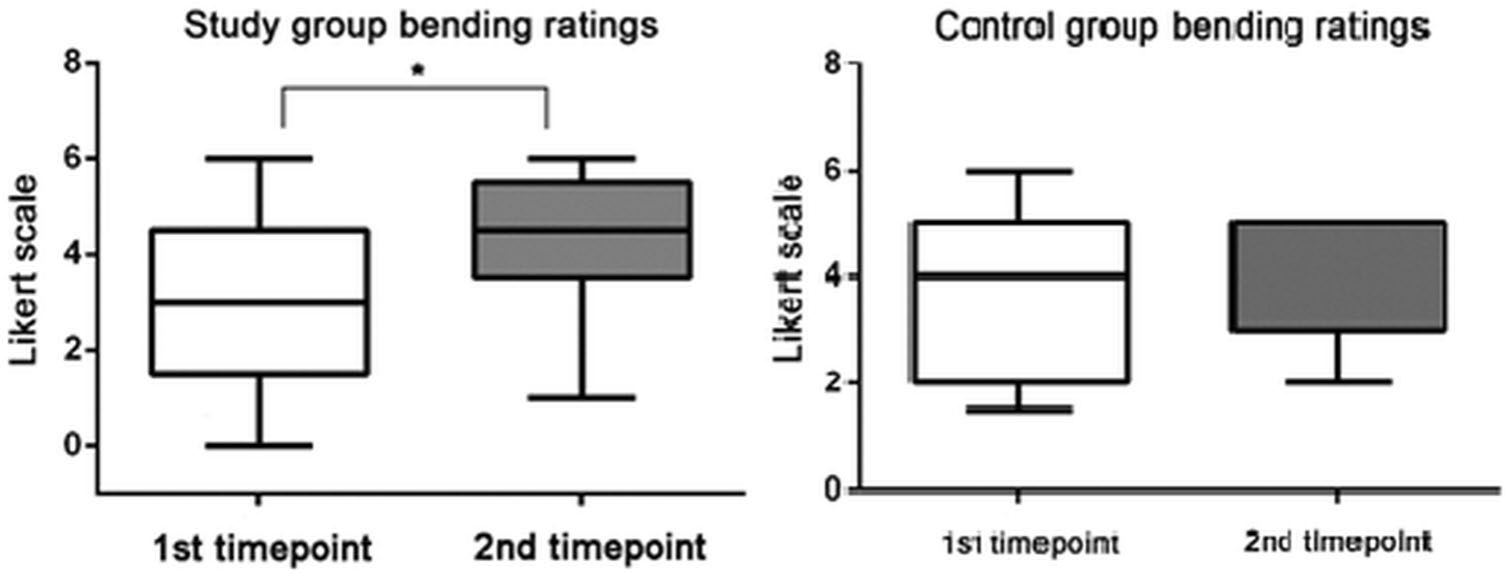
Evaluation with Likert scale of manual skills of the trained (study group) and non-trained (control) group of dentistry students. White and grey colors show the bending scores at 1st and 2nd time points, respectively. Significant difference between the scores of the first and second time points are marked by asterisks (*p < 0.05, Wilcoxon test). Whiskers are set to minimum and maximum, the horizontal line marks the median, whereas the box indicates the interquartile range (25–75%).

Figure 4 shows the results of the manual skills of the trained and control groups, which is assessed with the subjective method (Likert scale). The white and grey colors show the bending scores at the first and second time points, respectively. Significant difference between the scores of the first and second time points are marked by asterisks (*p < 0.05, Wilcoxon test). Whiskers are set to minimum and maximum, the horizontal line marks the median, whereas the box indicates the interquartile range (25–75%).

The comparison of pixels between the first and second time points, produced by the photo editing software showed similar results as the Likert scale evaluation. After a simple mathematical calculation the value of both measured parameters (wave and triangle) was closer to the original shapes in the trained group.

### MRI results

Table 2a,b show the CGM volume changes (in mm^3^) measured with MRI between the baseline and the follow-up for the both groups, separately.

**Table 2:** The results of the cerebellar grey matter volume changes study group (a) n=15, control group (b) n=15.

Cerebellar grey matter volume changes (between time point 2 and 1)	M (mm^3^)	SD (mm^3^)	Min (mm^3^)	Max (mm^3^)	p^a^
**(a) Study group (n = 15)**					
Right cerebellum	354.2	588.4	− 803.3	1228	0.0352
Left cerebellum	441.6	763.7	− 835.4	2156	0.0419
**(b) Control group (n = 15)**					
Right cerebellum	90.18	580.9	− 756.4	1273	0.5573
Left cerebellum	75.36	809	− 1182	1880	0.7237

The median (M) values (in mm^3^) of the cerebellar grey matter volume changes between time point 2 and 1 (after the training course and before it). Standard deviation (SD), minimum and maximum measured values and probability (p^a^) values are given for right and left cerebellum.

^a^Paired t-test.

**Figure 5:**
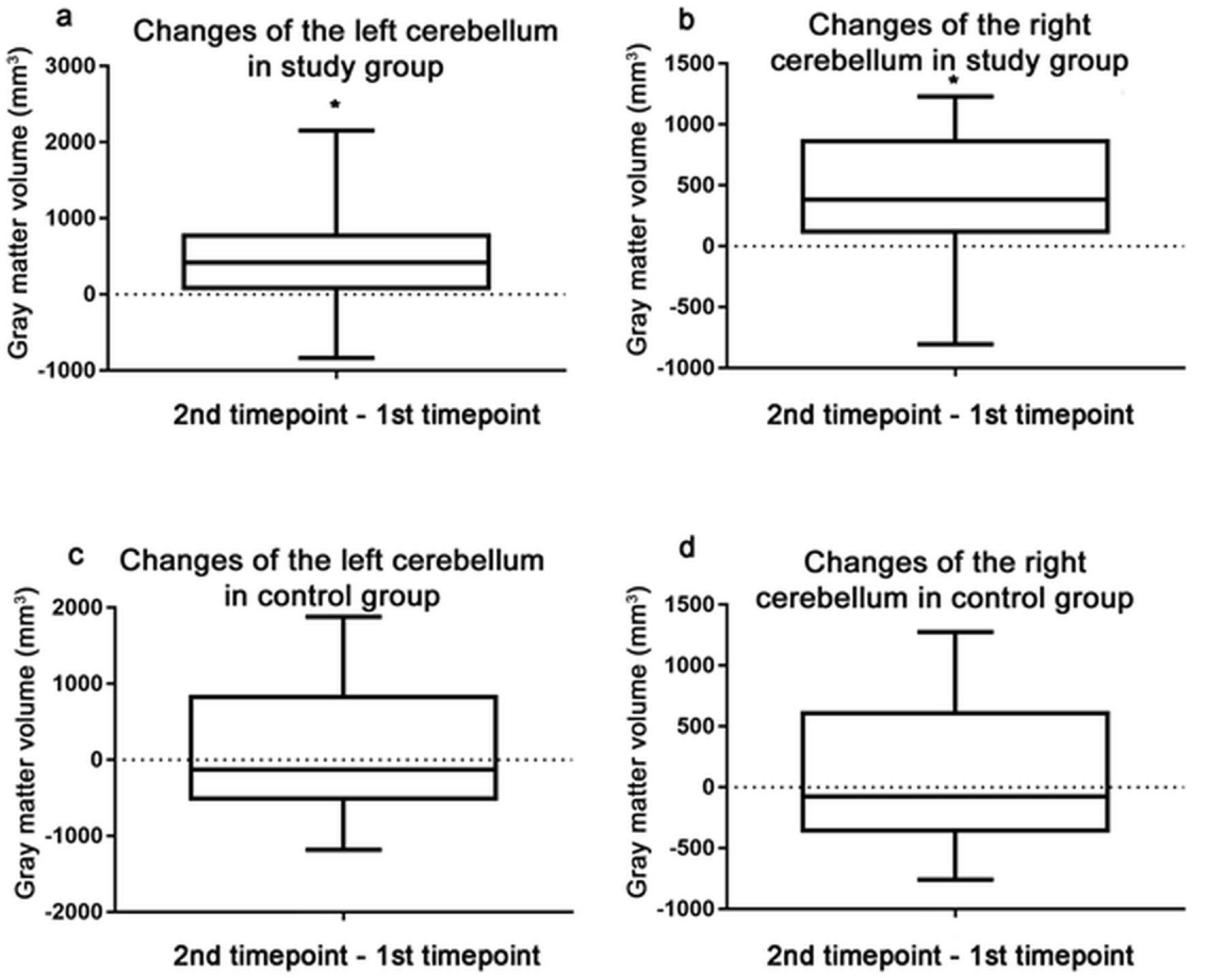
Grey matter volume changes of the trained (study) and non-trained (control) group of dentistry students. (**a**) Box and whisker plot of the left CGM volume change in the study group; (**b**) CGM volume changes of the right cerebellum in the study group; (**c**) CGM volume changes of the left cerebellum in the control group; (**d**) CGM volume changes of the right cerebellum in the control group. Whiskers are set at minimum and maximum, the horizontal line marks the median, whereas the box indicates the interquartile range (25–75%). Significant change can be seen in the CGM volume of the trained (study) group of students in both sides of the cerebellum (*p < 0.05).

The CGM volumes of the left and right cerebellum significantly increased in the study group, while no longitudinal changes were found in the control group (Table 2a,b, Fig. 5).

Figure 5 shows the Box and Whisker Plots of the CGM volume changes. Figure 5a gives the CGM volume changes of the left cerebellum in the study group; Fig. 5b shows the CGM volume changes of the right cerebellum in the study group, while Fig. 5c,d give the CGM volume changes of the left and right cerebellum in the control group, respectively.

The results of MRI measurements demonstrate a significant increase in the CGM volume in both sides of the cerebellum of the trained (study) group of students as a result of the manual skills training. Significant differences between the two time points are marked by asterisks (*p < 0.05, p paired t-test).

## Discussion

Our research aimed to prove the effectiveness of the training course, where the results revealed that this measurement can be used for this purpose. The increased CGM volume compared in the pre-training and post-training phases of the study were significant in the trained group. That result was simultaneously detected with the improvement of trained students’ manual dexterity following the training course. Parallel with our study, in general, the cerebellum may show a growth tendency after cognitive, IQ, memory and motoric training^52^.

The pixel differences provided by the photo editing software showed correlation with the results of the Likert scale. In the trained group, the value of each measured parameter, like pixels of wave and triangles were closer to the original shapes. The percentage determination of improvement was not possible in this study due to the limited number of participants. We are resolute to continue this research, and to measure how many percentages of pixel distances can really demonstrate an improvement of manual dexterity. The simple computer-based mathematical measurement designed by our team will be sufficient to evaluate the This simple computer based mathematical measurement will be sufficient to evaluate the student’s dexterity in the future.

Comparing the results of the first and second measurements, the study group showed improvement in dexterity however the control group was impaired.

The training course of our research consists of 10 sessions (10 weeks), which may explain why the time difference between our first and second measurements was so long. Since our results proved to be significant, it allows us to assume that a longer period of the training course (more than 10 weeks) or more frequent training sessions (more than 1 session a week) may lead to better performance.

In addition to our research, the observation of CGM volume changes was simultaneously done. We found a difference between volume changes of right and left CGM. The left CGM increased more than the right one, which means that the left side of right-handed students developed better. In healthy subjects, the dominant hand is more proficient at dexterity skills as compared to the non-dominant hand^52^. A further possible conclusion is that the non-dominant hand will show a profound improvement as opposed to the dominant hand which is already proficient. Taking this and the cerebello-spinal pathways into consideration, the left/non-dominant hand will show more improvement, demonstrated by a more significant increase in the left CGM volume. The increase in left CGM volume was due to a notable improvement in the non-dominant (in the present study the left) hand performance.

The training course was a comprehensive non-focused course on one or two of all skills, like hand–eye coordination or depth visualization. The training helped to develop both hands of the participants. Our findings concerning the cerebellum are confirmed by the results of other studies. Correlation between manual dexterity, grip force, and the size of both cerebellar cortices and age in healthy subjects had been reported^53^. In older people, no correlation between poor manual abilities and smaller cerebellar grey and white matter volumes were found^54^.

In a voxel-based morphometry study, a correlation was found between the right grey matter volumes of the Lobule VI of the cerebellum and the manual dexterity in healthy 14-year old children^55^. Our study focused on the total volume growth of cerebellar grey matter on both sides. The properties of our MRI scan device and the software did not allow to visualize smaller differences, so we did not detail the different parts of the cerebellar cortex.

Another report describes that CGM loss in the case of multiple sclerosis, results in decreased dexterity^56^. This research also proves that changes in CGM volume correlate with dexterity.

Another study proved that motor learning triggered changes in the plasticity of professional basketball players’ cerebellum. A significant difference was found in the size of the vermian lobules (VI-VII) when compared to the cerebellar white matter of professional basketball players and people not pursuing basketball^57^.

According to Doyon and Ungerleider’s model^36^, the cerebellum has a key role in brain plasticity during motor adaptation. Based on this theory, our syllabus of the skills developmental course for the first-year students was identified as training, activating slow learning. Up till now, the theory described by Doyon and Ungerleider has been applied in functional studies only. The model can be supplemented by volume analyses since volume changes appear to exhibit retention of the acquired skills during the slow learning process. The volume growth of the cerebellar grey matter may have been a result of the exercises from the developmental course.

The findings of our study suggest that the CGM volume significantly increases following the training course. The training resulted in significantly higher wire bending rates in the study group. Simultaneously we observed that Likert evaluation and the pixel based photo image measurement showed similar output: in our opinion they both are suitable for the measurement of dexterity. However, this study also has limitations: the group of participants was small, and the length of the training course was a quite short. Nowadays newly constructed software are used in the MR laboratories, which allows for detailed, voxel-based volumetry.

## Conclusion

We aimed to prove that training manual skills may promote the development of dexterity in undeveloped dentistry students. We hypothesized that this post-training improvement is measurable with MRI and HAM-Man dexterity tests. We used these examinations as longitudinal assessment tools, as they provided evidence of improvement in dental manual dexterity through improved wire bending and CGM volume changes. We hypothesized that CGM volume may increase by new motor learning, and this claim is supported by Doyon & Ungerleider’s model. Our results supported both predictions, since there has been a significant growth of the right and left CGM volume and simultaneously a significant improvement of manual dexterity in the study group.

Other important consequences of this investigation are related to the manual skills development courses offered by dental schools. The course was effective, leading to significant improvement in both measurements. Testing manual skills have an important role in universities where the admission procedure includes neither perception, nor dexterity skills assessment. Especially so for students whose manual dexterity test results are poorer. Finally, similarly to previous studies^58^, we can conclude that the training course has a key role in preparing students with poorer manual skills for the clinical module.
